# Premature Beats With QRS Morphology Identical to Sinus Rhythm: What Is the Mechanism

**DOI:** 10.1002/joa3.70386

**Published:** 2026-06-07

**Authors:** Chengye Di, Wenhua Lin

**Affiliations:** ^1^ First Department of Cardiology TEDA International Cardiovascular Hospital Tianjin China; ^2^ College of Clinical Cardiology Tianjin Medical University Tianjin China; ^3^ Cardiovascular Institute Tianjin University Tianjin China; ^4^ Chengde Medical University Chengde Hebei China

**Keywords:** atrioventricular nodal reentrant tachycardia, differential diagnosis, electrocardiogram, isolated beat, super‐ventricular contraction

## Abstract

This case illustrates a rare presentation of premature junctional contractions arising from the atrioventricular nodal region, particularly the slow pathway, mediated by non‐sustained reentrant activity. This mechanism does not result in sustained tachycardia but instead produces isolated echo beats.

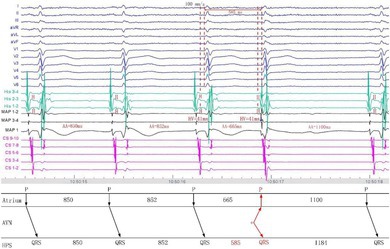

## 1 Case Presentation

**FIGURE 1:**
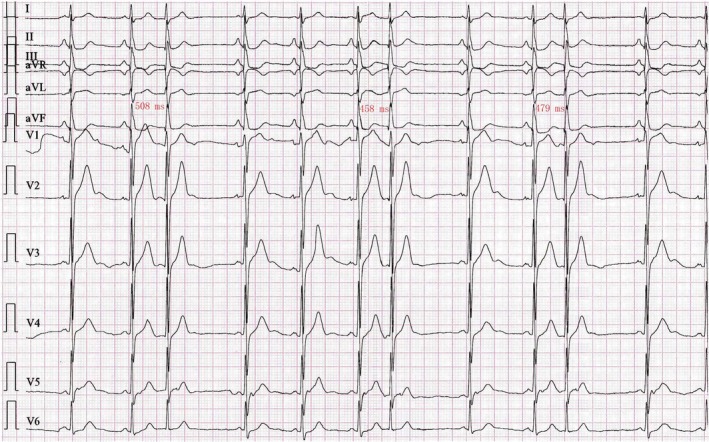
Twelve‐lead electrocardiogram demonstrating normal QRS morphology. Frequent narrow‐complex premature beats occur in a trigeminal or quadrigeminal pattern. These premature beats are not preceded by discernible P waves and exhibit coupling intervals ranging from 458 to 508 ms. The QRS morphology and duration of the premature beats are nearly identical to those observed during sinus rhythm (SR).

A 56‐year‐old man with no significant past medical history presented to our hospital with a 2‐year history of palpitations. A 12‐lead electrocardiogram (ECG) demonstrated normal QRS morphology, with a sinus cycle length of 800 ms, a PR interval of 129 ms, and a QRS duration of 102 ms. Frequent narrow‐complex premature beats were observed in a trigeminal or quadrigeminal pattern. These premature beats were not preceded by any discernible P waves and exhibited coupling intervals ranging from 458 to 508 ms. The QRS morphology and duration of the premature beats were nearly identical to those observed during sinus rhythm (SR) (Figure 1). What is the most likely mechanism of these premature beats, and what is the probable diagnosis?

## 2 Discussion

The ECG features shown in Figure 1 strongly suggest that ventricular activation originates very close to the normal His–Purkinje system, supporting a supra‐Hisian, Hisian, or infra‐Hisian origin near the His bundle. The differential diagnosis includes: (1) SR with dual ventricular response via dual atrioventricular nodal (AVN) pathways; (2) premature junctional contractions (PJCs), including those arising from the AVN region (particularly the slow pathway) and adjacent perinodal tissue; (3) premature Hisian contractions; (4) premature infra‐Hisian beats arising from the proximal fascicular system; and (5) premature atrial contractions (PACs) with rate‐dependent PR prolongation and concealed P waves fused within the preceding QRS complex or T wave.

**FIGURE 2:**
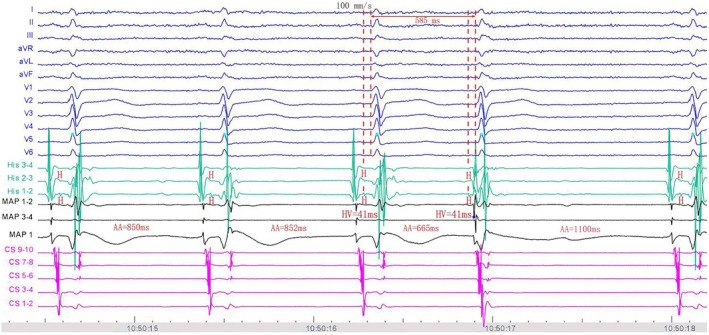
Intracardiac recordings show that the HV interval (41 ms), His electrogram morphology, and His activation sequence (proximal‐to‐distal) are identical during SR and premature beats, with no evidence of split His potentials. Additionally, atrial activation slightly precedes ventricular activation during the premature beat.

Premature beats without discernible preceding P waves and with QRS morphology identical to sinus beats raise concern for an origin at or very near the His bundle, where catheter ablation carries a risk of atrioventricular conduction injury [1]. In contrast, a supra‐Hisian origin (i.e., AVN or atrial) is generally associated with a higher ablation success rate and a lower risk of conduction block. During the electrophysiology study, ventricular pacing demonstrated normal ventriculoatrial decremental conduction. Atrial extrastimulation revealed dual AVN physiology, but no tachycardia was induced. As shown in Figure 2, the premature beat occurred with a coupling interval of 585 ms and was associated with atrial activation slightly preceding ventricular activation on both the His and CS recordings. This finding argues against PACs with rate‐dependent PR prolongation and concealed P waves completely fused within the preceding QRS complex or T wave.

**FIGURE 3:**
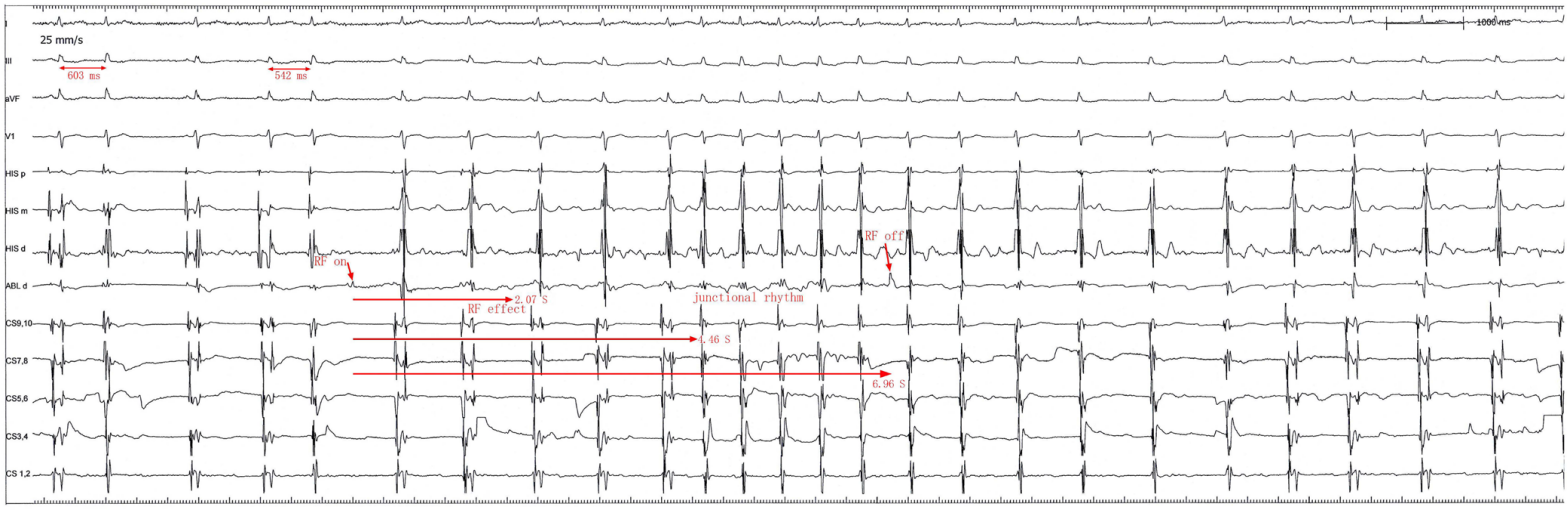
Radiofrequency ablation was delivered in the slow pathway region. Premature beats were abolished within 2.07 s of energy delivery, followed by the appearance of junctional rhythm after 4.46 s. Ablation was discontinued shortly thereafter, and SR gradually resumed without recurrence of premature beats.

Further analysis showed that the HV interval (41 ms), His electrogram morphology, and His activation sequence (proximal‐to‐distal) were identical during SR and premature beats, with no evidence of split His potentials. These findings effectively exclude an infra‐Hisian origin. Additionally, the observation that atrial activation slightly precedes ventricular activation during the premature beat further supports a supra‐Hisian origin rather than ventricular or infra‐Hisian origin. SR with dual ventricular response via dual AVN pathways was considered but is unlikely. This mechanism typically produces a relatively fixed coupling interval between the two ventricular activations. In contrast, the coupling intervals in this case were markedly variable (458–603 ms) (Figures 1, 2, 3), making this diagnosis improbable. Furthermore, SR with dual ventricular response is typically observed in the absence of an atrial echo for the slow pathway–conducted beat [2]. The presence of an atrial electrogram during the premature beats in this case further argues against this mechanism.

Radiofrequency ablation was delivered in the slow pathway region using a power‐controlled mode at 30 W. Premature beats were abolished within 2.07 s of energy delivery, followed by the appearance of junctional rhythm after 4.46 s. Ablation was discontinued shortly thereafter, and SR gradually resumed without recurrence of premature beats (Figure 3). Consolidation ablation was performed for a total of 120 s with junctional rhythm observed. Repeat atrial programmed stimulation no longer demonstrated dual AVN physiology. No premature beats were observed during a 1 h observation period, with or without isoproterenol infusion. Taken together, the findings are most consistent with PJCs arising from the AVN region, particularly the slow pathway. At 6‐month follow‐up, the patient remained free of recurrence.

Collectively, the ECG and electrophysiological characteristics observed in this case—including isolated premature beats, dual AVN physiology, atrial activation preceding ventricular activation, identical His electrogram morphology and activation sequence (proximal‐to‐distal) during SR and premature beats, absence of split His potentials, and immediate elimination of the premature beats following slow pathway modification—support the diagnosis of PJC arising from the AVN region, particularly the slow pathway, mediated by localized, non‐sustained reentrant activity. This mechanism does not result in sustained tachycardia but instead produces isolated echo beats. The variable coupling intervals further support a reentrant mechanism with limited stability and insufficient propagation to sustain tachycardia. This presentation falls within the broader spectrum of PJCs, given that the term “junctional” encompasses the AV node and adjacent perinodal tissue.

The observed target potentials are not consistent with those of typical focal premature atrial or ventricular contractions, in which bipolar recordings usually show the earliest atrial or ventricular activation preceding the surface P wave or QRS complex, and unipolar recordings demonstrate a sharp initial QS pattern. In contrast, during the premature beats in this case, the bipolar electrogram was not earlier than that recorded on the His catheter, and the unipolar electrogram exhibited a small initial r wave. The slow pathway target potential recorded during sinus rhythm was characterized by a small atrial component and a larger ventricular component, with an A/V ratio of approximately 1:2 to 1:3. The ablation strategy therefore mirrored that used for slow–fast AVNRT: although earliest activation was recorded near the His bundle, the target remained the slow pathway region, achieving a high success rate with minimal risk of atrioventricular conduction block.

Importantly, at the successful ablation site, no discrete preceding potentials (including low‐amplitude or dull components) were identified prior to the premature beats, as shown in Figure 2. Although the absence of a clearly identifiable preceding potential may appear to limit precise localization, this finding is not unexpected in the context of slow pathway–related activity. The slow pathway region is characterized by diffuse and anisotropic conduction properties, and electrical activity arising from this region often does not generate sharp, discrete pre‐potentials detectable by standard bipolar recordings. Instead, activation may be embedded within or overlap with the His electrogram or surrounding atrioventricular nodal signals, making separation difficult.

In summary, this case illustrates a rare presentation of PJCs arising from the AVN region, particularly the slow pathway, mediated by non‐sustained reentrant activity. This mechanism does not result in sustained tachycardia but instead produces isolated echo beats. Careful analysis of ECG features, intracardiac electrograms, HV interval, and His electrogram morphology and activation sequence were essential for accurate diagnosis and safe, effective ablation.

## Funding

This work was funded by the Tianjin Key Medical Discipline Construction Project (No. TJYXZDXK‐3‐035C).

## Disclosure

The authors have nothing to report.

## Conflicts of Interest

The authors declare no conflicts of interest.

## Data Availability

All raw data and recording during the case are available for review.

## References

[1] M. Alasti , S. Mirzaee , C. Machado , et al., “Junctional Ectopic Tachycardia (JET),” Journal of Arrhythmia 36, no. 5 (2020): 837–844. 33024461 10.1002/joa3.12410 PMC7532275

[2] C. W. Raphael and B. B. Pavri , “An Unusual Case of Non‐Reentrant Atrioventricular Nodal Tachycardia,” Journal of Cardiovascular Electrophysiology 33, no. 5 (2022): 1062–1066. 35229370 10.1111/jce.15426

